# Air Pollution Increased the Demand for Gym Sports under COVID-19: Evidence from Beijing, China

**DOI:** 10.3390/ijerph191912614

**Published:** 2022-10-02

**Authors:** Xin Dong, Shili Yang, Chunxiao Zhang

**Affiliations:** 1 School of Information Engineering, China University of Geosciences, Beijing 100083, China; 2 Beijing Meteorological Observation Centre, Beijing Meteorological Bureau, Beijing 100089, China

**Keywords:** air pollution, PM2.5 concentration, gym sports, spatial econometric model, COVID-19

## Abstract

Air pollution may change people’s gym sports behavior. To test this claim, first, we used big data crawler technology and ordinary least square (OLS) models to investigate the effect of air pollution on people’ gym visits in Beijing, China, especially under the COVID-19 pandemic of 2019–2020, and the results showed that a one-standard-deviation increase in PM2.5 concentration (fine particulate matter with diameters equal to or smaller than 2.5 μm) derived from the land use regression model (LUR) was positively associated with a 0.119 and a 0.171 standard-deviation increase in gym visits without or with consideration of the COVID-19 variable, respectively. Second, using spatial autocorrelation analysis and a series of spatial econometric models, we provided consistent evidence that the gym industry of Beijing had a strong spatial dependence, and PM2.5 and its spatial spillover effect had a positive impact on the demand for gym sports. Such a phenomenon offers us a new perspective that gym sports can be developed into an essential activity for the public due to this avoidance behavior regarding COVID-19 virus contact and pollution exposure.

## 1. Introduction

Many Chinese megacities are extremely polluted, and the problem of serious haze has aroused widespread concern among the public [1,2]. Air pollution not only significantly causes great harm to public health [3,4], it also affects people’s daily life [5,6]. Air pollution may inhibit outdoor physical activity, as people have engaged in avoidance behavior [7,8]. Some studies have demonstrated that air pollution has negative effects on the frequency of travel to indoor amenities such as restaurants, shopping areas and movie theatres [9,10] and outdoor venues such as public parks [11].

Notably, research related to indoor air pollution concentration in gyms is dominated by evidence from some developed countries, which generally show that people may inhale more pollutants while exercising in indoor gyms [12,13,14]. In cities of the developing world, however, the role of gym sports is under-researched. The existing literature, mainly focusing on the impact of air pollution on the frequency of gym physical activity, remains rare.

To further investigate how gym sports can be affected by air pollution and actual external environmental factors, this study focuses on the effect of air pollution under the COVID-19 pandemic. As is known, the COVID-19 pandemic is currently one of the greatest challenges to human life and health and economic development [15]. Beginning in 2020, starting in China, the coronavirus rapidly spread around the world due to globalization, which caused great harm to travel behavior [16]. The strictest social travel restrictions imposed by the Chinese government in response to the first outbreak of the pandemic included restricting all outdoor activities and closing all recreational facilities, including public indoor sports places [17,18]. Although lockdown restrictions started to lift in many cities around the world, regular pandemic prevention and control measures have remained unchanged in China, such as transport suspension [19] and 14 days of quarantine when traveling [20,21], referring to the post-pandemic era.

Recently, a significant number of studies have shown that the pandemic has reduced the frequency of people traveling to both outdoor physical activities [22,23] and indoor gym sports [24]. In addition, some scholars have found changes in air quality because of the travel restrictions during the COVID-19 pandemic [25,26]. Gym exercise behavior is affected by air pollution, and was especially affected during the COVID-19 pandemic [27]. Nevertheless, there is still a lack of studies that have analyzed the impact of air pollution on gym sports behavior under the impact of the pandemic in spatiotemporal dimensions.

**Figure 1:**
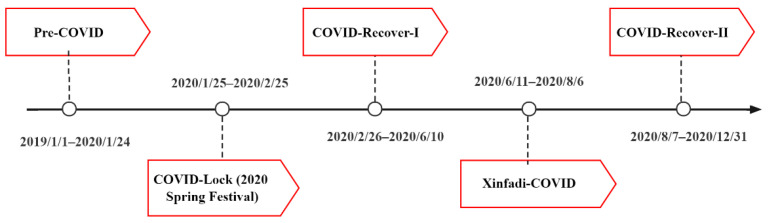
Timeline of five periods of the COVID-19 pandemic of Beijing, China.

The purpose of this study is to fill the gap aforementioned by examining this case in Beijing, China. Beijing, as the capital of China, with its vast territory, dense population and complex terrain, makes air pollution a serious threat [28]. Beijing was the second-largest city in China with gym exercisers in 2020, after Shanghai [29]. In this paper, we used big data crawling techniques and panel regression models to examine the effect of PM2.5 concentration conducted by land use regression (LUR) models [30,31] on gym visits during COVID-19 from 1 January 2019 to 31 December 2020. We divided 2019–2020 into five COVID-19 periods due to air pollution prevention levels published by the Chinese government, including the Pre-COVID period (the period before the pandemic, spanning from 1 January 2019 to 24 January 2020), COVID-Lock period (the period during the outbreak of the pandemic and the blockade of non-essential travel, spanning from 25 January to 25 February 2020), COVID-Recover-I period (the period during pandemic prevention and control downgrade, spanning from 26 January to 10 June 2020), Xinfadi-COVID period (the period of another outbreak of the pandemic in Xinfadi, spanning from 11 June to 6 August 2020) and COVID-Recover-II period (the period when the pandemic outbreak alert was lifted, spanning from 7 August to 31 December 2020), abbreviated as P1–P5 (Figure 1) [32]. Gym comments crawled from the platform “Meituan.com” were used to describe Beijing’s gym visits in P1–P5 of the COVID-19 pandemic.

In this study, we aimed to not only access the effect of air pollution on people’s gym visits under five different COVID-19 periods, but also to evaluate the spatial spillover effect of air pollution on the entire development of the gym industry from the perspective of a space–time analysis. Therefore, this study was designed to examine two research questions: (1) How does air pollution affect people’s demand for gym sports under COVID-19? (2) Is there a spatial spillover effect on the impact of air pollution on gym sports?

Our findings from the space–time analysis about the impact of air pollution on gym sports have contributed to the literature on air pollution and avoidance behavior. At the same time, it is necessary to pay attention to the impact of COVID-19 on people’s behavior regarding gym sports, which has been well documented as a new behavioral moderator regarding avoiding outdoor exposure of the virus when exercising.

## 2. Materials and Methods

### 2.1. Gym Data

**Figure 2:**
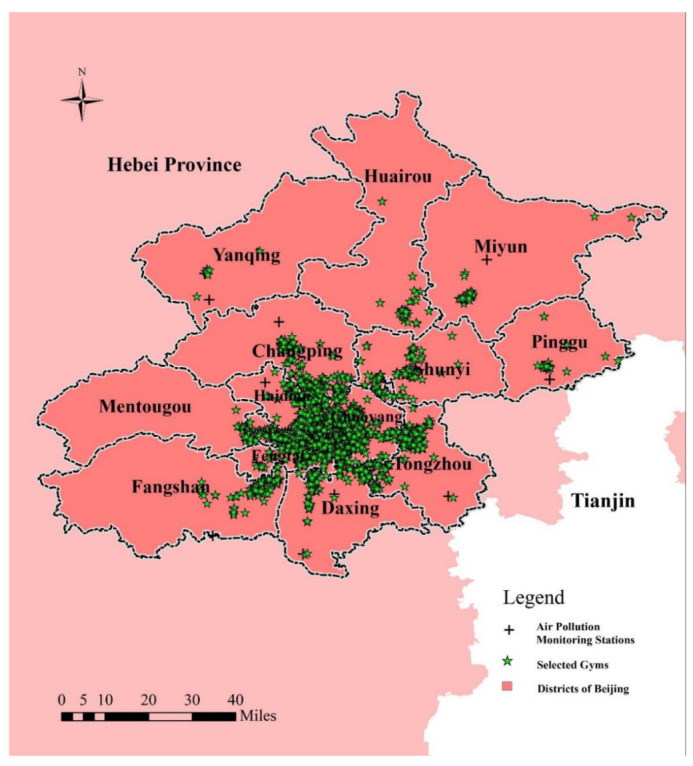
Study area. The spatial distribution of 2452 gyms of 16 administrative districts are indicated in Beijing, China.

This study aimed to test daily gym activity changes in Beijing that were associated with air pollution and climate conditions under the COVID-19 period. The study area and spatial distribution of 2452 gyms are shown in Figure 2. Because a lack of direct evidence on consumer mobility limited our ability to characterize local gym consumption conditions, to a certain degree, we used online consumption recorded data, which was used to represent the actual number of gym visits according to previous studies [9,33]. Therefore, we used big data crawler technology and crawled the data from the Chinese leading local lifestyle information and trading platform “Meituan.com” to describe Beijing residents’ gym activities. The information of each gym in Beijing included its precise location information and its attributes data, including users’ reviews, ratings and their per capita consumption from 1 January 2019 to 31 December 2020.

Finally, our analysis data included 2452 gyms with 144,904 comments. The comments data were merged according to the timeline of five different COVID-19 periods to describe Beijing’s gym visits during COVID-19. This study implicitly assumed that the probability of writing a review was not relevant to the air pollution level by using the count of reviews as a proxy for the count of gym visits.

### 2.2. PM2.5 Concentration Data

Hourly PM2.5 data in Beijing from 1 January 2019 to 31 December 2020 were obtained from the Beijing Municipal Environmental Monitoring Center [34]. Since 2013, there have been 35 environmental monitoring stations (EMSs) in Beijing (Figure 2). The botanical garden EMS was eliminated from the twelve urban EMSs in this study, and 34 EMSs were used to obtain hourly PM2.5 concentration in this study based on the Ambient Air Quality Standards (GB 3095-2012) [35]. We averaged the PM2.5 data based on the five different periods of the COVID-19 pandemic for the subsequent air pollution modeling.

### 2.3. Land Use Regression (LUR) Model

We used the LUR model to replace traditional spatial interpolation methods to obtain average PM2.5 concentrations of P1–P5 more accurately [30,31]. In our study, we divided the process into two steps as follows, including preparing predictor variables and building the LUR model.

#### 2.3.1. Predictor Variables

We used average PM2.5 concentrations of P1–P5 and independent variables to build LUR models, including land use, digital elevation model (DEM), meteorological variables, road length, population density, remote sensing PM2.5 data, normalized difference vegetation index (NDVI), aerosol optical depth (AOD) and points of interest (POI). These nine driving factors were selected on the basis of their significant impact on PM2.5 concentrations [36,37]. Overall, this study considered a total of 29 subcategories, which included 103 independent variables within the nine major independent variable categories.

As shown in Table 1, land use data, traffic information and POI data, the three major vector predictor variables, are included in the LUR model. Land use data were derived from Esri 2020 Land Cover with a spatial resolution of 10 m (https://livingatlas.arcgis.com/landcover/, accessed on 5 May 2021). Land use type was divided into cropland, woodland, grassland, water bodies, construction land, and unused land. The specific indicator of the land factor was the ratio of land area to buffer area (area of circular buffer).

Road data were obtained from OpenStreetMap (https://www.openstreetmap.org, accessed on 5 May 2021). The specific road factor indicator was the ratio of road length to buffer area.

Points of interest (POI) data were derived from Amap, by applying API based on category and keyword semantics (http://lbs.amap.com/api/webservice/guide/api/search/, accessed on 25 May 2021). Different POI categories in different buffer sizes represent different emission information with regard to PM2.5. In this study, we used four types of POI as pollutant emission sources, including bus stations, gas stations, polluted enterprises and Chinese restaurants. To reflect the impact of local emission sources and possible regional transmission, the buffer sizes for POI in this study were set from 100 to 7000 m [38].

As shown in Table 2, population data, geographical information, vegetation index, remote sensing data, meteorological data and aerosol optical depth data, the six major raster predictor variables, are included in the LUR model.

The population density data used in this study were based on Gridded Population of the World (GPW) from the Columbia University Socioeconomic Data and Application Center (CU2020) as raster data, with a resolution of 1 × 1 km [39].

Elevation data were obtained from the Advanced Spaceborne Thermal Emission and Reflection Radiometer Global Digital Elevation Model, version2 (ASTER GDEM v2) on the USGS Earth Explorer site, with a spatial resolution of 30 m (https://yceo.yale.edu/aster-gdem-global-elevation-data, accessed on 8 May 2021).

Normalized difference vegetation index (NDVI) data were provided by the MOD13A2 Version 6 product distributed by NASA EOSDIS Land Processes DAAC, which was a MODIS/Terra Vegetation Indices 16-Day L3 Global 1 km SIN Grid with a spatial resolution of 1 km (https://lpdaac.usgs.gov/products/mod13a2v006/, accessed on 12 May 2021). Data were calculated into mean data for the corresponding period of P1–P5 [40].

Remote sensing PM2.5 data were provided by the MODIS/Terra + Aqua Level 3 (L3) Yearly 0.01 degree gridded ground-level PM2.5 products in Eastern China (ECHAP_PM2.5_Y1K) from 2019 to 2020 (https://zenodo.org/record/4660858#.YU2JKux0IdU, accessed on 14 May 2021) [41,42].

Meteorological data were extracted from the ERA–Interim reanalysis dataset of European Centre for Medium-Range Weather Forecasts (ECMWF), with a spatial resolution of 0.125° × 0.125°. The data downloaded were daily means of 2-meter temperature data, U wind speed data, V wind speed data, boundary layer height data, surface pressure data and total precipitation data, which needed to be summed into mean data of P1–P5 by Python, prepared for later modeling [43].

Aerosol Optical Depth (AOD) data were obtained from the MCD19A2 Version 6 data product distributed by NASA EOSDIS Land Processes DAAC, which was a Moderate Resolution Imaging Spectroradiometer (MODIS) Terra and Aqua combined Multi-angle Implementation of Atmospheric Correction (MAIAC) Land Aerosol Optical Depth (AOD) gridded Level 2 product produced daily at 1 km pixel resolution. (https://lpdaac.usgs.gov/products/mcd19a2v006/, accessed on 15 May 2021) [44].

#### 2.3.2. Building the LUR Model

A five-step backward method was adopted for fitting the LUR model [45]. For all available potential predictor variables, bivariate correlation analysis with average PM2.5 concentrations of P1–P5 were conducted first. Second, the predictor variables were sorted by adjusted R^2^. Third, other variables (Pearson correlation coefficient R ≥ 0.7) with high relevance to the highest ranked variables in each subcategory were removed. Fourth, all the remaining predictor variables and PM2.5 concentrations were entered into the stepwise linear regression model to obtain the multiple linear regression equation. Finally, the significance level (*p* value < 5%) and variance inflation factor (VIF < 4) of each predictor variable were checked to confirm the variables’ significance levels and to ensure no issues of multicollinearity. The leave-one cross-validation (LOOCV) and 10-fold CV method were chosen to evaluate the predictive capacity of the model. From the cross-validation, the R^2^ and root mean squared error (RMSE) were used to evaluate and compare the predictivity of the model [46,47].

### 2.4. Statistical Analysis

**Figure 3:**
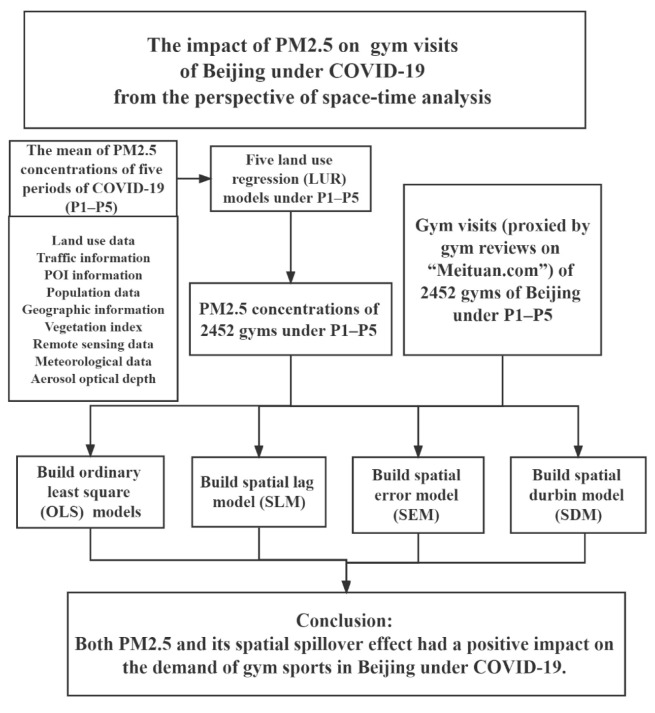
Theoretical framework model of this study.

Figure 3 shows the overall theoretical framework used for assessing the effect of PM2.5 concentration on gym visits under COVID-19.

#### 2.4.1. Building Ordinary Least Squares (OLS) Model

First, we used an OLS panel regression model to test how the count of the gym visits varies as a function of air pollution and climate conditions.

(1) $$NUM_{it}=\alpha_{0}+\alpha_{1}PM2.5_{it}+\alpha_{2}W_{it}+\alpha_{3}X_{it}+T_{t}+\gamma_{i}+\varepsilon_{it},$$

where $NUM_{it}$ and $PM2.5_{it}$ represent the comments, reviews, and PM2.5 concentrations of gym *i* on COVID-19 period *t*. $W_{it}$ represents weather conditions, including gym mean temperature and precipitation of P1–P5. $X_{it}$ is used as a control variable, including for residents’ gym ratings and per capita consumptions. $T_{t}$ and $\gamma_{i}$ are used to control for COVID-19 wave-fixed effects and gym area-fixed effects. $\varepsilon_{it}$ is the random error term. The standard errors are clustered by individual. To avoid collinearity with the PM2.5 data generated by the LUR model, the average temperature and precipitation data of P1–P5 used in Equation (1) are based on other meteorological data obtained from 18 standard meteorological stations at the Beijing Meteorological Informational Center, including hourly temperature and precipitation.

Second, we defined the variable COVID-19 to denote the number of confirmed cases in P1–P5 waves into Equation (1) to generate Equation (2).

(2) $$NUM_{it}=\alpha_{0}+\alpha_{1}COVID\text{-}19_{t}+\alpha_{2}PM2.5_{it}+\alpha_{3}W_{it}+\alpha_{4}X_{it}+T_{t}+\gamma_{i}+\varepsilon_{it},$$

where $COVID\text{-}19_{t}$ represents the cumulative number of confirmed cases of COVID-19 in P1–P5. Coefficient $\alpha_{2}$ reflects the effect of PM2.5 concentration on gym visits under the background of the COVID-19 pandemic, which is expected to be consistent with coefficient $\alpha_{1}$ in Equation (1). The meanings of other characters are as the same as the above model.

Third, a robustness check was used to replace the PM2.5 variable with PM2.5 data obtained from the Kriging interpolation method of the MEP PM2.5 monitoring sites and that from the United States Embassy and Consulates to verify the association of actual PM2.5 concentration with gym visits in P1–P5 of the COVID-19 pandemic [48].

#### 2.4.2. Building Spatial Econometric Models

To begin, we used Moran’s Index to examine whether there was a spatial autocorrelation relationship or not between gym visits in P1–P5 of the COVID-19 pandemic [49] as Equation (3).

(3) $$Moran^{\prime }s\;I=\frac{N}{\sum_{ij}^{N}w_{ij}}\;\frac{\sum_{ij}^{N}w_{ij}\;\left(x_{i}-\bar{x}\right)\left(x_{j}-\bar{x}\right)}{\sum_{i=1}^{N}{\left(x_{i}-\bar{x}\right)}^{2}}$$

with *i* ≠ *j*, where $x_{i}\;$ is the interest variable in gym *i*, $\bar{x}\;$ is the variable mean value, *N* is the number of observations, and *w_ij_* is the spatial weight. The Moran index varies between −1 and 1. A value close to 1 indicates the presence of clusters, and a value close to −1 indicates spatial dispersion in the data. If the value is 0, then there is no spatial autocorrelation.

If the spatial factor was ignored or classified as a random disturbance term, the results obtained by the OLS modeling would have errors. Therefore, when analyzing the impact of PM2.5 on gym visits, spatial econometric models need to be considered [50].

Then, spatial factors were taken into account in the spatial lag model (SLM), which emphasized the spatial spillover effect of the local gym visits to the number of exercisers in other nearby gyms. The SLM model is expressed formally as Equation (4):

(4) $$NUM_{it}=\alpha +\rho \;\sum_{j=1}^{n}w_{ij}\;NUM_{jt}+\beta_{1}COVID\text{-}19_{t}+\beta_{2}PM2.5_{it}+\beta_{3}W_{it}+T_{t}+\gamma_{i}+\varepsilon_{it}$$

where ρ is the influence degree of the explained variable with spatial lag on the explained variable, $w_{ij}$ represents the weight matrix, *β* is the degree of influence of each explanatory variable (COVID-19, PM2.5 and weather situations) on gym visits, and the meanings of other characters are the same as the above models.

Furthermore, the spatial error model (SEM) measured the error impact of local influential factors on the gym visits themselves and the impact of these influential factors on other gyms in Beijing. The SEM model is expressed formally as Equation (5):

(5) $$NUM_{it}=\alpha +\beta_{1}COVID\text{-}19_{t}+\beta_{2}PM2.5_{it}+\beta_{3}W_{it}+T_{t}+\gamma_{i}+\varepsilon_{it}$$

$$\varepsilon_{it}=\lambda \;\sum_{j=1}^{n}w_{ij}\;\varepsilon_{jt}+\mu_{it}\;,\;\mu_{it}\;\sim \;N\left[0,\;\delta^{2}I\right]$$

where *λ* represents the spatial error correlation coefficient, $\mu_{it}$ is the error term that satisfies the OLS assumption, and the meanings of other characters are the same as the above models.

Lastly, the spatial Durbin model (SDM) reflects the degree of agglomeration of gym visits in a single gym, the level of agglomeration of gym visits in adjacent gyms, and the impact of the overall gym visits on the development of the gym industry in Beijing. The SDM model is expressed formally as Equation (6)

(6) $$NUM_{it}=\alpha +\rho \;\sum_{j=1}^{n}w_{ij}\;NUM_{jt}+\beta_{1}COVID\text{-}19_{t}+\beta_{2}PM2.5_{it}+\beta_{3}W_{it}+\gamma \;w_{ij}COVID\text{-}19_{t}+\gamma \;w_{ij}PM2.5_{t}+\gamma \;w_{ij}W_{t}+T_{t}+\gamma_{i}+\varepsilon_{it}$$

where $w_{ij}COVID\text{-}19_{t}$, $\;w_{ij}PM2.5_{t}$ and $w_{ij}W_{t}$ represent the spatial lag COVID-19, PM2.5 and weather situations (temperature and precipitation) of the average observation value of the adjacent gyms. *γ* is the spatial correlation coefficient, and the meanings of other characters are the same as the above models.

## 3. Results

### 3.1. PM2.5 Concentration Estimates from the LUR Model

**Figure 4:**
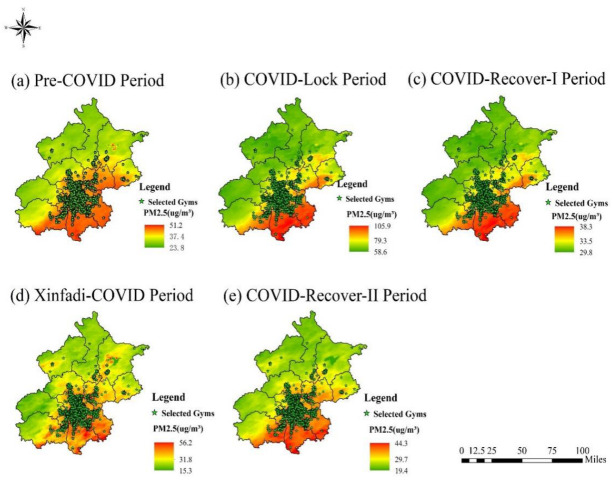
Spatial distribution of simulation results of PM2.5 concentrations in Beijing in P1–P5.

The results of the five LUR model simulations were strongly correlated with the independent in situ PM2.5 values based on the leave-one cross-validation (LOOCV) and 10-fold CV results on the pandemic scale of P1–P5 (Table 3). After the final regression models were obtained, regular 1 × 1 km grids were generated and predicted PM2.5 concentrations were calculated at the grid points. Then, Kriging interpolation was conducted to generate PM2.5 concentration distribution simulation maps of Beijing in P1–P5 (Figure 4). Finally, we extracted the corresponding PM2.5 concentration values of all gyms in P1–P5 as PM2.5 exposure concentrations in P1–P5 of the COVID-19 pandemic, preparing for the follow-up study of the effect of air pollution on gym visits under COVID-19.

### 3.2. The Impact of PM2.5 Concentration on Gym Visits under COVID-19

As shown in Table 4, we used a series of OLS models to present specifications that include progressively more controls from Model 1 to 3. Model 1 only controls for time-fixed effects of five different COVID-19 waves, adding in area-fixed effects in Model 2, gym attributes control variables in Model 3, and finally the level and squared terms of PM2.5 in Model 4, which were used to explore the nonlinearity of the relationship between PM2.5 concentration and gym visits. The standardized coefficients of PM2.5 from Model 1 to 3 steadily decreased (0.150, 0.148 and 0.119), showing that PM2.5 was significantly positively associated with gym visits. Furthermore, precipitation was significantly negatively associated with gym visits with an effect that was 18.05 in Model 4 after controlling for time-fixed effects, area-fixed effects and gym attribute variables. Model 4 exhibits a nonlinear positive relationship between PM2.5 concentration and gym visits; thereby, our study mainly focused on the linear effect of PM2.5 on gym visits under COVID-19.

Moreover, the positive impact of PM2.5 grew from 0.119 in Model 3 of Table 4 to 0.171 in Model 1 of Table 5, which meant that such a positive effect increased to a 0.051 standard deviation when introducing the COVID-19 variable into Model 3. Notably, we found that COVID-19 was negatively associated with gym visits (−0.0272 in Model 1 of Table 5), which could be explained by Chinese pandemic control measures and people’s high awareness of pandemic prevention under the COVID-19 pandemic.

In our robustness check, PM2.5 from the Kriging interpolation method in Model 2 of Table 5 was not statistically significantly associated with gym visits. However, the positive impact of PM2.5 from the US Embassy and Consulates grew to almost 14 times of that in Model 3 (2.341 versus 0.171). This might be due to the fact that there is only one PM2.5 monitoring site in the US Embassy and Consulates of Beijing, leading to an overestimation of the positive impact of pollution on gym visits. The correlation between any two of the three PM2.5 indicators was as high as 0.96. We found that the coefficient of precipitation was significant and negative.

When considering the spatial autocorrelation, the z-value and *p* value of the overall Moran’s Index for the gym visits showed that at the 1% significant level (Table 6), the number of gym exercisers had a significantly positive spatial autocorrelation among different gyms in Beijing, indicating that there was an obvious agglomeration phenomenon among the gym crowds.

Because of the spatial autocorrelation, spatial econometric models needed to be taken into account, which included the spatial lag model (SLM), spatial error model (SEM) and the spatial Durbin model (SDM).

As shown in Table 7, PM2.5 concentrations were positively associated with gym visits in the OLS model, SLM model and SEM model after controlling for both fixed effects, with coefficients of 0.171, 0.205 and 0.197, respectively, which effectively illustrated that PM2.5 had a positive effect on gym visits under COVID-19. Notably, the standardized coefficients of COVID-19 from the OLS model, SLM model, SEM model and SDM model were similar (−0.027, −0,027, −0.028 and −0.024), which indicated that the COVID-19 variable itself inhibited people’s gym sports behavior.

In addition, the spatial autocorrelation coefficients ρ of both the SLM model and the SDM model and the spatial autoregressive coefficient λ of the error term in the SEM model were all significantly positive (0.078, 0.076 and 0.078), indicating that gyms with large gym visits could increase the number of exercisers of surrounding gyms and drive the development of the gym industry of Beijing, which was consistent with the previous analysis of the Moran’s Index.

Compared with the SLM model and SEM model, we adopted the SDM model to carry out the following analysis due to LR test rejecting the null hypothesis, which showed that the SDM model was more reliable and stable. In order to analyze the spatial effect of each influencing factor more concretely, we decomposed the SDM model to obtain the direct effect (the influence of PM2.5 on local gyms), indirect effect (the impact of PM2.5 on other adjacent gyms), and total effect (the impact of PM2.5 on all gyms of Beijing). The specific results are listed in Table 8.

As shown in Table 8, the direct effect of PM2.5 was not significant, while the indirect and total effects of PM2.5 were both significantly positive. It was proven that every 1% increase in PM2.5 promoted an increase in gym visits at adjacent gyms by 0.394%, and an increase in gym visits in all of Beijing by 0.512%. This phenomenon could be explained because the air pollution had a spatial heterogeneity, that was to say, people with exposure to higher pollution concentrations would refuse to go outside for exercise, while people with relatively lower exposure to pollution nearby were more willing to travel to gyms. This phenomenon needs to be discussed in future studies.

Furthermore, the direct effect and the total effect of COVID-19 on fitness were significantly negative, while the indirect effect was not significant. In reality, there was a negative impact on gym fitness for all people when the pandemic was severe enough.

## 4. Discussion

### 4.1. Discussion of Main Findings

Given the high level of air pollution in Beijing, China, its air pollution dynamics offered the opportunity to test how the demand for gym sports for this consumer city was affected by pollution in the context of the intertwined impact of air pollution and the COVID-19 pandemic.

Our study chose Beijing as the case to investigate how air pollution influenced people’s demand for gym sports, focusing on the effect of PM2.5 on the frequency of traveling to gyms during the COVID-19 pandemic. Our OLS model results showed that PM2.5 was positively associated with gym visits, which was opposite of previous research studies showing that air pollution was negatively associated with both outdoor physical activity [51,52] and indoor leisure places such as restaurants and shopping areas [9,10]. Such a positive impact was larger when considering the influence of the COVID-19 pandemic. One reason to explain this result was that people could not wait to exercise and participate in recreational activities after the strict travel mandates of the COVID-19 pandemic [16,18]. Therefore, gym indoor physical activity gave them a chance to ignore the harmful effects of both air pollution and COVID-19 virus contact when exercising. We also observed that COVID-19 reduced the demand for gym sports, which was consistent with our expected results [24]. Whether the accelerating effect of the COVID-19 pandemic on the positive impact of air pollution on gym sports could trade off its own negative impact on gym sports or not still needs more research and exploration. Future research should explore the interweaving effects of the climate and social environment on people’s sports behavior.

Furthermore, in the section about measuring PM2.5 concentrations in the P1–P5 periods under COVID-19, five LUR models were constructed to accurately evaluate the long-term exposure of gym crowds while traveling to the gym. Seasonality (i.e., spring, summer, autumn and winter) was not included as a variable in the analysis of this section because collinearity existed between season and ambient temperature after we controlled for seasonality in this study. In the following robust check, we replaced the PM2.5 data from the LUR models with that obtained through the Kriging interpolation method of the MEP PM2.5 monitoring sites and that from the US Embassy and Consulates [48]. When taken together, the reported positive effects of air pollution on gym visits derived from OLS models in our study may be pronounced and solid.

Lastly, our study employed spatial econometric models, including SLM, SEM and SDM to explore the spatial pattern of the gym visits in Beijing. We concluded that there was an obvious agglomeration phenomenon among the gym crowds, in other words, the increase in gym visits at local gyms would lead to an increase in gym visits at other nearby gyms, thereby making it possible to facilitate rapid development of the entire gym industry of Beijing. Furthermore, we used the SDM model to obtain the direct effect, indirect effect and total effect to verify the spatial spillover influence of PM2.5 on gym visits in Beijing under COVID-19. The results showed that PM2.5 had a positive spatial spillover effect on gyms visits of nearby gyms and on the all gyms in Beijing. COVID-19 was negatively associated with gyms visits of all gyms, which was consistent with the above OLS models.

### 4.2. Implications for Pollution Health and Gym Sports Research

To the best of our knowledge, the present study provides evidence of the relationship between air pollution and gym sports under COVID-19. Gyms, as a combination of sports and indoor leisure consumption activity, are becoming relatively safe and comfortable places for people to exercise and avoid pollution exposure. Furthermore, gym exercise played an important role in improving people’s fitness and enriching their daily life under the rigorous pandemic prevention and control measures. This phenomenon offers us a new perspective that promoting gym use can produce considerable health benefits mainly from less outdoor pollution and from COVID-19 virus exposure, as well as from increased levels of physical activity.

Constructing more public indoor fitness facilities can be a good option to promote the vigorous development of mass sports. However, the local government and companies should consider the unintended impacts that constructing and operating new gyms may have on local air pollution, for example emissions from construction activities [53], onsite energy generators or increased demand for electricity from local power plants [54]. Furthermore, previous research has shown that people are more willing to pay for greenspace under air pollution [55]. It is essential to ensure that enough greenspace is provided in indoor fitness facilities to prevent each individual from pollution exposure and to create a comfortable and fresh exercise environment, which are beneficial for human mental health [56].

Taking into account the situation of the COVID-19 pandemic and its prevention, the government also needs to guarantee enough personal exercise space and avoid dangerous problems caused by big occupancy on the basis of increasing indoor gym infrastructure. For the issue of charging for private or public gyms, the government should improve the free or low-cost opening subsidy policy for public sports facilities and promote the opening of facilities to all groups of people.

Therefore, the local government should encourage gym sports and promote the benefits of indoor fitness after the comprehensive consideration of the impact of various influencing factors on people’s indoor fitness, especially under the COVID-19 pandemic. As a result, more and more citizens are aware of the importance of physical activity under the intertwined influences of both the pandemic and air pollution, which will continuously improve the participation rate of national fitness in the future.

### 4.3. Research Limitations and Future Research Agenda

Our study has important limitations. First, given the high level of air pollution in Beijing, China, our data come from 2452 gyms in Beijing, which may lead to possible selection bias, and we need to be careful in generalizing this bias to other contexts. Further research in other cities and countries is needed to cross-validate the external validity of our quantitative findings. Second, we consider PM2.5 as the main source of pollutants in this study, ignoring other urban pollutants, such as PM_10_, NO_2_ and SO_2_ [57]. Third, we used PM2.5 generated from LUR models under five COVID-19 periods to represent the pollution exposure concentration of gym crowds. However, this lacks measurements of the specific time of their gym visits and their personalized pollution exposure risk from different commutes to the gym, which are expected to be resolved in follow-up research. Finally, it is unclear whether exercise in a gym produce similar health benefits as outdoor sports. We need to consider multifaceted environmental attributes to possibly exert the health effects at different spatial scales of gyms, which requires further research.

## 5. Conclusions

Using big gym datasets of Beijing in 2019–2020, we documented that air pollution had a positive impact on residents’ gym visits (proxied by gym reviews on “Meituan.com”) under COVID-19. Such a positive impact was larger when considering the influence of the COVID-19 pandemic. From the perspective of space analysis, PM2.5 had a positive spatial spillover effect on the development of the whole gym industry in Beijing under COVID-19.

Overall, this study offers us a new perspective that gym sports can be developed into an essential activity for the public due to the avoidance of COVID-19 virus contact and pollution exposure. Furthermore, this study may provide useful information for a number of relevant stakeholders and policymakers and may be informative for city and gym infrastructure planning that aims to promote better public health outcomes.

In the future, we need to consider the health hazards caused by large gatherings of gym crowds and make some reasonable suggestions about gym management, for example, making sure there are enough greenhouses in gyms and increasing gym facilities on the basis of causing as little air pollution as possible and ensuring enough personal exercise space under a pandemic prevention environment. In addition, due to the lack of direct evidence on people’s movement, we had to use online consumption data to replace the original activity track data. In follow-up research, we need to measure personalized exposure pollution, such as different levels of air pollution exposure by different commutes to gyms and real-time indoor pollution concentration of indoor gyms.

## Figures and Tables

**Table 1:** Description of the vector predictor variables in the LUR models of P1–P5.

No.	Predictor Variables	Abbreviations	Unit	Buffer Size
Land use				
1	Cultivated land	Cul_xx	m^2^	100, 300, 500, 1000, 3000, 5000
2	Forest	For_xx	m^2^	100, 300, 500, 1000, 3000, 5000
3	Grass land	Gra_xx	m^2^	100, 300, 500, 1000, 3000, 5000
4	Waterbody	Wat_xx	m^2^	100, 300, 500, 1000, 3000, 5000
5	Built-up area	Bui_xx	m^2^	100, 300, 500, 1000, 3000, 5000
6	Unused land	Unu_xx	m^2^	100, 300, 500, 1000, 3000, 5000
Traffic information				
7	Trunk road length	Tru_xx	m	100, 300, 500, 1000, 3000, 5000
8	Primary road length	Pri_xx	m	100, 300, 500, 1000, 3000, 5000
9	Secondary road length	Sec_xx	m	100, 300, 500, 1000, 3000, 5000
10	Railroad length	Rai_xx	m	100, 300, 500, 1000, 3000, 5000
POI information				
11	Bus station number	POI1_xx	-	100, 300, 500, 1000, 3000, 5000, 7000
12	Gas station number	POI2_xx	-	100, 300, 500, 1000, 3000, 5000, 7000
13	Polluted enterprise number	POI3_xx	-	100, 300, 500, 1000, 3000, 5000, 7000
14	Chinese restaurant number	POI4_xx	-	100, 300, 500, 1000, 3000, 5000, 7000
15	Distance to the nearest bus station	D_bus	m	NA
16	Distance to the nearest gas station	D_gas	m	NA
17	Distance to the nearest polluted enterprise	D_pol	m	NA
18	Distance to the nearest Chinese restaurant	D_res	m	NA

**Table 2:** Description of the raster predictor variables in the LUR models of P1–P5.

No.	Predictor Variables	Abbreviations	Unit	Original Spatial Resolution
Population				
1	Population density	Pop	people/m^2^	1 km
Geographic information				
2	Elevation	DEM	m	30 m
Vegetation index				
3	NDVI	NDVI	-	1 km
Remote sensing data				
4	CHAP_PM2.5	CHAP	μg/m^3^	1 km
Meteorological data				
5	Boundary layer height	BLH	m	0.125°
6	2 m temperature	T2M	K	0.125°
7	Total precipitation	TP	mm	0.125°
8	Surface pressure	SP	10^6^ Pa	0.125°
9	10 m u-component of wind	U10m	m/s	0.125°
10	10 m v-component of wind	V10m	m/s	0.125°
Aerosol optical depth				
11	Optical_Depth_047	AOD_047	-	1 km

**Table 3:** Summary of the final LUR models for PM2.5 in P1–P5.

Model	Variable	Coefficient	Std. Error	T Value	*p* (>|t|)	VIF	Global Statistics
Pre-COVID PM2.5 (μg/m^3^)	Intercept	2.34	5.552	0.421	0.676	NA	Adjusted R^2^ = 0.68; LOOCV R^2^ = 0.68 RMSE = 3.10 μg/m^3^; 10-fold CV R^2^ = 0.68; RMSE = 3.72 μg/m^3^.
	CHAP	0.079	0.014	7.192	0.000	1.039	
	Cul_3000	4.174 × 10^−7^	1.363 × 10^−7^	3.063	0.004	1.039	
COVID-Lock PM2.5 (μg/m^3^)	Intercept	5.868	8.578	0.684	0.499	NA	Adjusted R^2^ = 0.73; LOOCV R^2^ = 0.73 RMSE = 5.12 μg/m^3^; 10-fold CV R^2^ = 0.73; RMSE = 6.26 μg/m^3^.
	CHAP	2.197	0.265	8.274	0.000	1.002	
	Cul_300	4.23 × 10^−4^	9.5 × 10^−5^	3.063	0.000	1.002	
COVID-Recover-I PM2.5 (μg/m^3^)	Intercept	20.925	2.973	7.039	0.000	NA	Adjusted R^2^ = 0.64; LOOCV R^2^ = 0.64 RMSE = 1.95 μg/m^3^; 10-fold CV R^2^ = 0.64; RMSE = 2.34 μg/m^3^.
	CHAP	0.398	0.093	4.297	0.000	1.048	
	Tru_500	1198.099	345.514	3.468	0.002	1.053	
	Cul_300	1.17 × 10^−4^	3.3 × 10^−5^	3.577	0.001	1.017	
	Gra_100	−0.001	2.52 × 10^−4^	−2.545	0.017	1.033	
Xinfadi-COVID PM2.5 (μg/m^3^)	Intercept	2.032	6.088	0.334	0.741	NA	Adjusted R^2^ = 0.73; LOOCV R^2^ = 0.73 RMSE = 3.36 μg/m^3^; 10-fold CV R^2^ = 0.73; RMSE = 3.97 μg/m^3^.
	AOD_047	0.066	0.013	5.24	0.000	1.674	
	POI3_7000	0.215	0.046	4.723	0.000	2.854	
	Pri_500	−2640.034	445.498	−5.926	0.000	1.017	
	POI2_7000	−0.19	0.058	−3.274	0.003	3.94	
	Wat_100	−49.1	1.72 × 10^−4^	−2.615	0.014	1.101	
COVID-Recover-II PM2.5 (μg/m^3^)	Intercept	8.407	4.257	1.975	0.057	NA	Adjusted R^2^ = 0.54; LOOCV R^2^ = 0.54 RMSE = 2.89 μg/m^3^; 10-fold CV R^2^ = 0.542; RMSE = 3.61 μg/m^3^
	AOD_047	0.079	0.014	5.505	0.000	1.044	
	Cul_1000	3.7 × 10^−6^	8.407	4.257	0.045	1.044	

**Table 4:** Estimated associations between PM2.5 and gym visits from the OLS models.

Dependent Variable: Gym Comments	Model 1	Model 2	Model 3	Model 4
PM2.5	0.150 ***	0.148 ***	0.119 ***	0.366 ***
	(0.000)	(0.000)	(0.000)	(0.008)
(PM2.5)^2^	-	-	-	−0.00165 *
	-	-	-	(0.065)
Precipitation	4.509	−20.01 ***	−18.05 ***	−19.17 ***
	(0.153)	(0.000)	(0.000)	(0.000)
Temperature	1.486 ***	−0.0322	0.0259	-0.0561
	(0.001)	(0.897)	(0.916)	(0.823)
Constant	11.16 *	36.81 ***	0.327	30.61 ***
	(0.075)	(0.000)	(0.939)	(0.000)
COVID-19 Wave FEs	Yes	Yes	Yes	Yes
Gym FEs	No	Yes	Yes	Yes
Gym Controls	No	No	Yes	Yes
Observations	12,260	12,260	12,260	12,260
Participant number	2452	2452	2452	2452

Note: We suppressed the coefficients on control variables to conserve space. The 95% confidence intervals are based on heteroscedastic robust standard errors. *** *p* < 0.001; * *p* < 0.01.

**Table 5:** Estimated associations between PM2.5 and gym visits in P1–P5.

Dependent Variable: Gym Comments	Model 1	Model 2	Model 3
COVID-19	−0.0272 ***	−0.0246 ***	−0.0246 ***
	(0.000)	(0.000)	(0.000)
PM2.5	0.171 ***	-	-
	(0.000)	-	-
PM2.5 Kriging	-	0.00305	-
	-	(0.952)	-
PM2.5 US Embassy	-	-	2.341 ***
	-	-	(0.000)
Precipitation	−19.14 ***	−17.14 ***	−17.04 ***
	(0.000)	(0.000)	(0.000)
Temperature	0.116	0.0712	0.0707
	(0.626)	(0.771)	(0.773)
Constant	−2.985	4.568	−94.63 ***
	(0.479)	(0.307)	(0.000)
Gym FEs	YES	YES	YES
COVID-19 Wave FEs	YES	YES	YES
Gym Controls	YES	YES	YES
Observations	12,260	12,260	12,260
Participant Number	2452	2452	2452

Note: We included time-fixed effects of five different COVID-19 waves, area-fixed effects and gym attributes control variables in all Models. In Model 2 and 3, we replaced the PM2.5 variable with PM2.5 data obtained from the Kriging interpolation method of the MEP PM2.5 monitoring sites and that from the US Embassy and Consulates, as mentioned in Materials and Methods. The 95% confidence intervals are based on heteroscedastic robust standard errors. *** *p* < 0.001.

**Table 6:** Moran’s Index of gym comments in P1–P5.

Dependent Variable: Gym Comments under P1–P5	*I*	*z*	*p* Value
Pre-COVID	0.181 ***	6.137	0.000
COVID-Lock	0.062 ***	2.159	0.015
COVID-Recover-I	0.100 ***	3.394	0.000
Xinfadi-COVID	0.102 ***	3.490	0.000
COVID-Recover-II	0.122 ***	4.229	0.000

Note: *** *p* < 0.001.

**Table 7:** Four regression model results in P1–P5.

Dependent Variable: Gym Comments under P1–P5	OLS	SLM	SEM	SDM
COVID-19	−0.027 ***	−0.027 ***	−0.028 ***	−0.024 ***
	(0.000)	(0.000)	(0.000)	(0.008)
PM2.5	0.171 ***	0.205 **	0.197 **	0.110
	(0.000)	(0.017)	(0.025)	(0.233)
Precipitation	−19.14 ***	−14.128	−13.897	5.397
	(0.000)	(0.223)	(0.253)	(0.757)
Temperature	0.116	0.164	0.196	0.347
	(0.626)	(0.671)	(0.614)	(0.376)
ρ	-	0.078 ***	-	-
	-	(0.000)	-	-
λ	-	-	0.078 ***	0.076 ***
	-	-	(0.000)	(0.000)
Gym FEs	YES	YES	YES	YES
COVID-19 Wave FEs	YES	YES	YES	YES
Observations	12,260	12,260	12,260	12,260
Participant Number	2452	2452	2452	2452

Note: *** *p* < 0.001; ** *p* < 0.05.

**Table 8:** Direct effect, indirect effect and total effect of the SDM model.

Dependent Variable: Gym Comments under P1–P5	Direct	Indirect	Total
COVID-19	−0.024 ***	−0.003	−0.027 ***
	(0.006)	(0.788)	(0.001)
PM2.5	0.118	0.394 ***	0.512 ***
	(0.210)	(0.003)	(0.000)
Precipitation	4.025	−45.799 **	−41.773 ***
	(0.810)	(0.028)	(0.007)
Temperature	0.367	−1.412 *	-1.045
	(0.328)	(0.065)	(0.215)
Gym FEs	YES	YES	YES
COVID-19 Wave FEs	YES	YES	YES
Observations	12,260	12,260	12,260
Participant Number	2452	2452	2452

Note: *** *p* < 0.001; ** *p* < 0.05; * *p* < 0.01.

## Data Availability

The data that support the findings of this study are available from the corresponding author upon reasonable request.
